# A comparative analysis of video vision transformers on word-level sign language datasets

**DOI:** 10.1371/journal.pone.0341909

**Published:** 2026-02-05

**Authors:** Jubayer Ahmed Bhuiyan Shawon, Md Kamrul Hasan, Hasan Mahmud

**Affiliations:** Systems and Software Lab (SSL), Department of CSE, Islamic University of Technology (IUT), OIC, Board Bazar, Gazipur, Bangladesh; Lamar University, UNITED STATES OF AMERICA

## Abstract

Sign Language Recognition (SLR) involves the automatic identification and classification of sign gestures from images or video, converting them into text or speech to improve accessibility for the hard-of-hearing community. In Bangladesh, Bangla Sign Language (BdSL) serves as the primary mode of communication for many individuals with hearing loss. This study fine-tunes state-of-the-art video transformer architectures VideoMAE, ViViT, and TimeSformer on BdSLW60, a small-scale BdSL dataset with 60 frequent signs. We standardized the videos to 30 FPS, resulting in 9,307 user trial clips. To evaluate scalability and robustness, the models were also fine-tuned on BdSLW401, a large-scale dataset with 401 sign classes. Additionally, we benchmark performance against public datasets, including LSA64 and WLASL. Data augmentation techniques such as random cropping, horizontal flipping, and short-side scaling were applied to improve model robustness. To ensure balanced evaluation across folds during model selection, we employed 10-fold stratified cross-validation on the training set of the BdSLW60 dataset, while signer-independent evaluation was carried out using held-out test data from unseen users U4 and U8. Results show that video transformer models significantly outperform traditional machine learning and deep learning approaches. Performance is influenced by factors such as dataset size, signer appearance, frame distribution, frame rate, and model architecture. Among the models, the VideoMAE variant (MCG-NJU/videomae-base-finetuned-kinetics) achieved the highest accuracies 96.9% on the frame rate corrected BdSLW60 dataset and 81.04% on the front-facing signs of BdSLW401 demonstrating strong potential for scalable and accurate BdSL recognition.

## 1 Introduction

More than 430 million people worldwide, including approximately 34 million children, experience some form of hearing loss constituting about 5% of the global population. Alarmingly, this number is projected to double by 2050, underscoring the urgent need for scalable and effective communication solutions for the Deaf and hard-of-hearing community [1]. Sign languages, which rely on intricate combinations of hand gestures, movements, postures, and facial expressions, serve as the primary mode of communication for many of those affected by hearing loss [2]. However, communication remains a significant challenge, as most hearing individuals lack fluency in sign language. This communication barrier is further exacerbated by the scarcity, high cost, and limited accessibility of professional sign language interpreters, thereby impeding the social inclusion and daily interaction of Deaf individuals [3].

Sign Language Recognition (SLR) aims to bridge this gap by leveraging computer vision and machine learning to automatically interpret sign language gestures [4]. SLR approaches are typically divided into two categories: isolated recognition, which focuses on identifying individual signs or fingerspelling frames, and continuous recognition, which interprets temporal sequences of signs to form phrases or sentences [5,6]. While continuous SLR must contend with ambiguous sign boundaries and temporal segmentation, isolated SLR operates at the gloss level, where each video contains exactly one sign.

A variety of isolated sign language datasets such as AUTSL, LSA64, WLASL, BosphorusSign22k, and LSM have propelled research forward in languages like Turkish, Argentinian and American Sign Language [7–11]. In contrast, studies on isolated Bangla Sign Language (BdSL) remain limited by data scarcity and resource constraints. The subtle intra-class variations and fine-grained hand movements in BdSL further complicate classification tasks, making high-accuracy recognition challenging.

Early efforts in SLR relied on traditional machine learning methods with hand-crafted features, which often struggled with scalability and robustness. The advent of deep learning (DL) particularly convolutional and recurrent neural networks has substantially improved visual recognition performance across many domains [12,13], including isolated SLR [14,15]. However, naive attempts to train attention-based DL architectures from scratch on BdSL data have yielded suboptimal performance, underscoring the need for more effective model adaptation strategies [16].

Recently, transformer-based architectures notably video transformers [17–19] and detection transformers [20] have demonstrated strong capabilities in modelling spatiotemporal dependencies for word-level sign recognition. In particular, a recent comparative study [21] evaluated the performance of VideoMAE and TimeSformer on the WLASL100 dataset, highlighting the effectiveness of transformer models in this domain. However, there remains a lack of comprehensive comparative analyses that account for key factors influencing performance across word-level sign language datasets varying in scale and complexity.

Transfer learning [22–27], wherein pre-trained models are fine-tuned on domain-specific data, has emerged as a powerful tool for boosting accuracy in data-scarce scenarios. Key considerations in transfer learning include the selection of which layers to transfer and whether to freeze or fine-tune them [28].

While prior studies have focused primarily on recognizing Bangla sign letters and numerals [29–31], limited work has addressed word-level BdSL recognition using modern deep learning techniques such as EfficientNet-B3, attention-based transformers, and BiLSTM [16,32,33]. To the best of our knowledge, no prior research has applied video transformers to word-level BdSL recognition.

In this work, we explore the potential of pre-trained video transformers such as VideoMAE, ViViT, and TimeSformer for isolated BdSL recognition. These models are fine-tuned separately on both the BdSLW60 and the larger BdSLW401 datasets. Their performance is evaluated to assess generalization and scalability across datasets of varying sizes. While earlier efforts in BdSL have predominantly focused on static fingerspelling or character-level recognition, our approach targets dynamic, word-level recognition using spatiotemporal modeling. Furthermore, we extend our evaluation across other sign language datasets to investigate the robustness of these models under varying frame rates, and class distributions, thereby addressing key challenges in low-resource sign language recognition.

In short, the key contributions of our work are as follows:

1. We examine accuracy fluctuations resulting from FPS correction and improve performance by introducing variations in uniformly chosen frames.
2. We present the first large-scale benchmark of transformer-based video models (VideoMAE, ViViT, TimeSformer) fine-tuned on isolated BdSL datasets, and conduct a comprehensive comparative analysis across other public sign language datasets (LSA64, WLASL100, WLASL2000) to evaluate performance trends and dataset-specific challenges.
3. We analyze the impact of frame imbalance, FPS (25, 30, 60) in small (BdSLW60, LSA64, WLASL100) to large-scale datasets (BdSLW401, WLASL2000), per-class sample size, model architecture, and signer appearance on the performance of video transformers.

We organise this work as follows: Sect 2 provides a survey of relevant literature and analyses contemporary methodologies and their deficiencies. Sect 3 outlines the proposed architecture, dataset preparation, an overview of the video transformers, configurations, and the fine-tuning process. Sect 4 outlines experimental results and performance evaluation. Sect 5 concludes the paper by delineating prospective research avenues.

## 2 Literature review

Sign language is a rich form of visual communication that encompasses both manual elements (hand movements, posture, position) and non-manual aspects (facial expressions, head gestures), assessed by traditional machine learning and deep learning methods [34]. Models such as support vector machines (SVM), hidden markov models (HMM), artificial neural networks (ANNs), and multilayer perceptrons (MLPs) have been applied with handcrafted feature extraction methods, including DCT, PCA, LDA, SURF, and SIFT, to improve classification accuracy [35,36].

Several early efforts reported promising results. Al-Rousan et al. [37], for instance, used HMM and DCT to classify 30 Arabic signs, achieving 94.2% accuracy in signer-independent settings. Similarly, Fagiani et al. [38] applied HMM to 147 Italian signs, although the accuracy reached only 50%, suggesting the need for more expressive models. Deep learning (DL) approaches emerged as a more powerful alternative, automating feature extraction and enabling end-to-end learning. A BiLSTM-based model with DeepLabv3+ hand segmentation achieved 89.5% accuracy for 23 Arabic signs [7], while Fatmi et al. [39] found that ANN and SVM outperformed HMM in American Sign Language (ASL) recognition.

More recently, hybrid DL models have improved word-level recognition. Masood et al. [40] integrated inception-based CNNs with RNNs for real-time Argentine sign language detection. Similarly, ResNet50 combined with LSTM was used in [41] for Persian sign videos, achieving accurate recognition across 100 signs. In another approach, spatial-temporal features from pretrained networks were fused, achieving 98.97% accuracy on the Montalbano dataset [42]. Additionally, [43] a multimodal fusion technique using quantized depth images with skeleton-based LSTM and depth-based CRNN models achieved 90.82% and 89.21% accuracy for 14 and 28 gestures, respectively, on the DHG-14/28 dataset, and 93.81% and 90.24% on the SHREC-2017 track dataset. CNN-transformer combinations have also shown promise: Shin et al. [44] used such a hybrid to attain 88.8% accuracy on a 77-class Korean Sign Language (KSL) dataset.

Research on Bangla Sign Language (BdSL) has also progressed. Raihan et al. [45] introduced channel-wise attention using squeeze-and-excitation blocks in a CNN model, reaching 99.86% accuracy on the KU-BdSL alphabet dataset with a lightweight model optimized for mobile deployment. In another study, Begum et al. [31] utilized quantization on YOLOv4-Tiny with LSTM to achieve 99.12% accuracy on the BdSL49 dataset. Other works have employed pose-based recognition using tools like OpenPose and Mediapipe. For example, [46] used OpenPose for Flemish sign recognition, while [16,32,47,48] explored Mediapipe-based keypoints for dynamic body part tracking in Arabic and Bangla sign language recognition.

Attention mechanisms and transformer architectures have also made significant contributions. Rubayeat et al. [16] applied attention-based BiLSTMs with SVM to BdSLW60, achieving 75.1% accuracy, and Hasan et al. [32] used an attention-based transformer for BdSL word-level recognition. These advances illustrate the growing utility of attention-based models, especially when combined with pose or spatiotemporal features. Knowledge transfer and transfer learning have further improved performance, as shown by [49], who used MobileNetV2 with transfer learning to achieve 95.12% accuracy on CSL-500 and a 2.2% word error rate on CSL-continuous. Follow-up studies on BdSL also leveraged pretrained models like DenseNet201 and MobileNetV2 to boost recognition accuracy [50,51].

In parallel, video transformers have emerged as powerful tools for sign language recognition due to their ability to model complex temporal and spatial dependencies. For example, a study in [19] evaluated several video transformer models, including VideoMAE and SVT, on the large scale WLASL2000 dataset—demonstrating the effectiveness of pretraining and fine-tuning in large-scale sign language recognition. Similarly, Detection Transformers (DETR) have been adapted to identify signs from RGB video inputs [21]. Beyond sign language, BERT has been combined with TimeSformer to improve the classification of short video clips [52], and the ViViT model has been applied to detect mild cognitive impairment from video sequences, showing competitive performance [53].

Recognizing the limitations in existing BdSL research particularly the underutilization of transformer-based video models this work explores isolated BdSL word recognition using state-of-the-art video transformers. We fine-tune models pretrained on the Kinetics-400 action recognition dataset, which includes gestures and movements similar to isolated sign language actions. Our focus is on improving recognition performance from raw RGB videos using models such as VideoMAE, ViViT, and TimeSformer. Additionally, we analyze critical design factors such as frame distribution, frame rate (FPS), and model architecture that influence recognition outcomes.

## 3 Methodology

This study addresses the classification of isolated BdSL signs, with particular attention to the challenges posed by limited resources and the inherent complexity of sign language datasets. To tackle these issues, we fine-tune transformer-based video classification models to effectively capture temporal patterns in sequential data. In this work, three models are trained on BdSLW60, and to assess scalability, BdSLW401 is used marking its first use as a benchmark. Their performance is rigorously evaluated and compared with results from other benchmark datasets, including WLASL and LSA64, highlighting the models’ generalization and robustness.

### 3.1 Framework overview and dataset preparation

**Fig 1:**
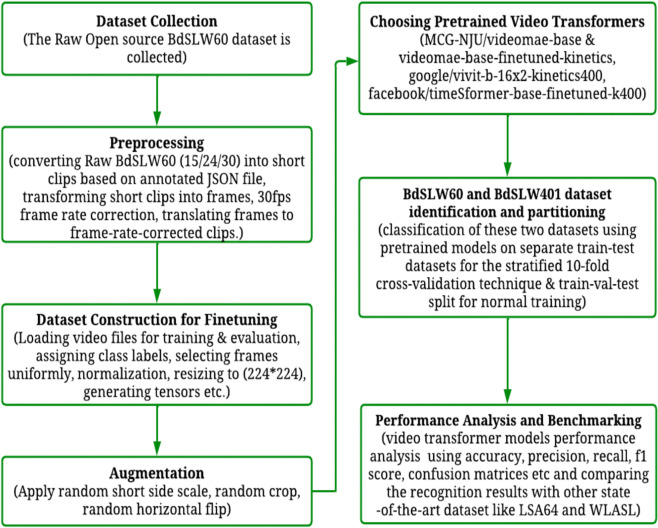
Architecture of frame rate-corrected dataset construction, recognition, and benchmarking. This figure illustrates the end-to-end workflow adopted for isolated sign language recognition across diverse datasets. The process begins with data collection and preprocessing, including clip segmentation, frame extraction, and frame rate correction. After preparing the dataset through frame selection, resizing, and tensor generation, data augmentation techniques such as horizontal flipping are applied to improve model generalization. Pretrained video transformer models (VideoMAE, ViViT, and TimeSformer) are then fine-tuned and evaluated using standard metrics, including accuracy, precision, recall, and F1-score. The results are compared with existing state-of-the-art approaches to assess model performance.

Fig 1 presents the overall architecture of the proposed approach, illustrating the complete pipeline from dataset acquisition through preprocessing, model training, and ultimately, evaluation.

**Fig 2:**
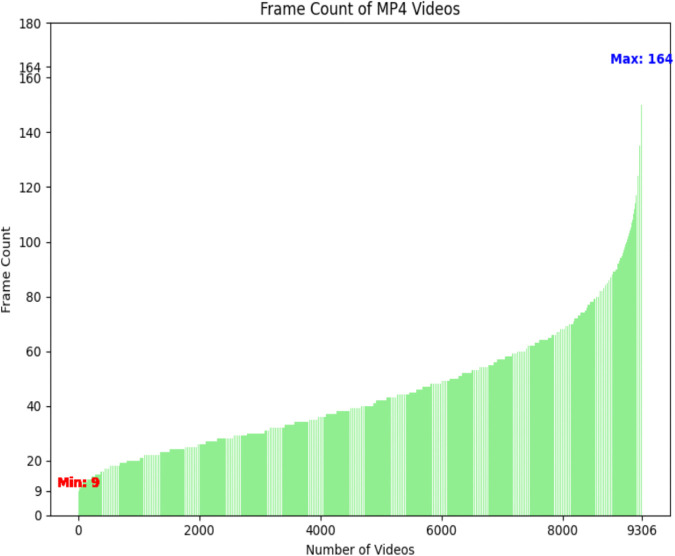
Frame Count vs Number of short clips of BdSLW60 dataset. This figure displays the distribution of frame counts across 9,307 short video clips in the BdSLW60 dataset. Each bar represents the frame count of an individual MP4 clip, sorted in ascending order. The minimum and maximum frame counts are highlighted in red and blue, respectively—ranging from 9 to 164 frames per clip.

The BdSLW60 dataset [16] is employed in this study, with dedicated preprocessing applied to ensure robust training and evaluation. The dataset, sourced from Kaggle, comprises Bangla sign language videos recorded by 18 individuals. Each sample includes raw video footage and corresponding gloss annotations provided in JSON format. The videos were originally recorded at varying frame rates—15, 24, and 30 FPS—but were standardized to 30 FPS for consistency. The JSON annotations facilitated the extraction of individual frames, resulting in clip lengths ranging from 9 to 164 frames per gloss. As shown in Fig 2, most samples contain fewer than 130 frames. In addition to BdSLW60, we employed the BdSLW401, WLASL100, WLASL2000, and LSA64 datasets to comprehensively evaluate the performance and generalizability of our approach.

### 3.2 Video processing

Determining the appropriate sample rate—defining the duration of each video clip used in training—requires a thorough understanding of the frame distribution across the dataset. This step is particularly critical for transformer-based models, which rely on fixed-length input sequences due to the patch embedding mechanism. The clip duration is calculated using the following equation: $𝒞=\frac{𝒩\times 𝒮}{ℱ}$, where 𝒞 is the clip duration, 𝒩 is the number of frames to sample, 𝒮 is the sample rate, and ℱ is the frame rate (fps).

To mitigate the impact of class imbalance and ensure robust performance evaluation, we adopted a 10-fold stratified cross-validation procedure. Frame rate-corrected (FRC) samples were employed to partition the dataset into training, validation, and testing subsets for video processing tasks, or into training and testing sets for stratified folding. The number of frames per clip was determined based on model-specific requirements and a predefined sampling rate. Clips with fewer frames than required were temporally padded to match the duration of the longest clip, ensuring input consistency. Additionally, all video frames were resized to 224×224 pixels and standardized to maintain uniformity and compatibility across model architectures.

### 3.3 Augmentation for training

During the data augmentation phase, we applied several techniques to improve dataset diversity and model generalization. These included random cropping, horizontal flipping, and short-side scaling. The isolated sign videos were subsequently trained in batches using three fine-tuned transformer-based models: VideoMAE [54], ViViT [55], and TimeSformer [56]. To optimize frame-level feature extraction, we utilized image processors specifically designed for each pretrained model architecture. Model performance was continuously monitored on both validation and test sets throughout the training process. Upon completion, the best-performing model checkpoints were uploaded to the Hugging Face (HF) repository for public access and reproducibility.

### 3.4 Model configurations

We employed three transformer architectures for training and evaluation, with one model implemented in two distinct configurations, as summarized in Table 1. All models were pretrained on the Kinetics-400 human action recognition dataset [57], which enables the transfer of temporal and spatial pattern recognition capabilities applicable to isolated sign classification. Fine-tuning on the BdSLW60 and auxiliary datasets led to high accuracy on both test and validation splits, demonstrating the models’ ability to effectively generalize to the target task.

**Table 1:** Comparison of different video transformer models and their architecture details.

Model Name	MCG-NJU/videomae-base & -finetuned-kinetics	google/vivit-b-16x2-kinetics400	facebook/TimeSformer-base-finetuned-k400
image_size	224	224	224
initializer_range	0.02	0.02	0.02
intermediate_size	3072	3072	3072
num_attention_heads	12	12	12
num_channels	3	3	3
num_frames	16	32	8
num_hidden_layers	12	12	12
patch_size	16	-	16
tubelet_size	2	[2, 16, 16]	2
hidden_act	gelu	gelu_fast	gelu
decoder_hidden_size	384	-	-
decoder_intermediate_size	1536	-	-
decoder_num_attention_heads	6	-	-
decoder_num_hidden_layers	4	-	-
use_mean_pooling	true	-	-
**Trainable Parameters**	94.2M & 86.5M	86M	121M

This table compares the architectural configurations of three video transformer models VideoMAE, ViViT, and TimeSformer all fine-tuned on Kinetics datasets. Each model uses a 224×224 input size and 12 attention heads, but differs in frame usage: VideoMAE uses 16 frames, ViViT uses 32, and TimeSformer uses 8. Only VideoMAE includes a decoder with specific hidden size and attention layers. While all have 12 hidden layers and 3072 intermediate size, their activation functions and patch/tubelet sizes vary. TimeSformer has the largest number of trainable parameters (121M), followed by VideoMAE and ViViT.

#### 3.4.1 Video Mask Auto Encoder—VideoMAE architecture.

The idea of VideoMAE is found in Image-MAE [58], where the image masking strategy is described for better accuracy gain in recognition tasks. VideoMAE [59] is a simple masked video autoencoder with an asymmetric encoder-decoder design. To efficiently handle sampled frames, it introduces cube embedding and high-ratio tube masking, where only a small subset of visible tokens is encoded, and the decoder reconstructs the masked tokens for self-supervised learning [54].

Tube masking is implemented to address video redundancy, employing a high masking ratio (90-95%) to avert information loss and improve reconstruction, especially in low-motion segments. The model incorporates an encoder that exclusively handles unmasked cubes and a streamlined decoder. Video segments are subjected to cube embedding, with merely 5-10% of tokens inputted into the encoder. The model subsequently forecasts the masked tokens by reducing the discrepancy between target and projected clips. Tube masking surpasses alternative techniques by employing a uniform mask across the frames. The disordered tokens are reconstituted, and absent tokens are acquired through backpropagation. A compact decoder reconstructs video segments to assess performance. By encoding fewer tokens and using joint space-time attention [55] with a ViT backbone [60], this method shortens the time needed for training.

#### 3.4.2 Video Vision Transformer—ViViT architecture.

Researchers have extended ViT [60], originally developed for image classification, to create transformer-based models for video classification [55]. These models use self-attention in the encoder to capture long-range contextual relationships within video sequences. Earlier approaches tackled this challenge using deep 3D CNNs [61,62] and by incorporating self-attention in later layers [63–65].

ViViT improves the Vision Transformer by integrating attention variations specifically designed for video data. It employs solely the encoder of the transformer [66], analysing video clips $𝒱\in {\mathbb{R}}^{T\times H\times W\times C}$ transformed into token sequences $\tilde{Z}\in {\mathbb{R}}^{n_{t}\times n_{h}\times n_{w}\times d}$. Two techniques—uniform frame sampling and tubelet embedding—convert videos into non-overlapping tokens, with tubelet embedding more efficiently integrating the temporal dimension. A tubelet possesses dimensions *t* × h×w, with the quantity of tubelets along each axis defined as $n_{t}=\left\lfloor \frac{T}{t}\right\rfloor$, $n_{h}=\left\lfloor \frac{H}{h}\right\rfloor$, and $n_{w}=\left\lfloor \frac{W}{w}\right\rfloor$. Following the incorporation of positional embeddings, tokens are input into the encoder, where self-attention scores are calculated. The ViViT model comprises 12 encoder layers and 12 attention heads. Four attention mechanisms are examined:

1. Spatio-temporal Attention: Employs Multi-Head Self-Attention (MSA) [66] across all tokens, leading to quadratic complexity.
2. Factorised Encoder: Distinguishes between spatial and temporal tubelet processing, reducing floating-point operations while preserving global context.
3. Factorised Self-Attention: Executes attention initially in the spatial domain, followed by the temporal domain, preserving Model 2’s complexity while enhancing parameter efficiency.
4. Factorised Dot-Product Attention: Distributes attention heads evenly over spatial and temporal domains, optimising complexity and parameter quantity.

Ultimately, a multilayer perceptron (MLP) forecasts class labels during the training process. This method improves efficiency while preserving robust performance in video representation learning.

#### 3.4.3 TimeSformer architecture.

TimeSformer is a video categorization model that operates without convolution, utilizing a Vision Transformer. It separates video frames into N separate patches, uses learnable positional encoding, and a 12-layer transformer encoder to process them. The initial token, ${𝒵}_{\left(0,0\right)}^{\left(0\right)}$, functions as a classification token, with patch embeddings articulated as:

$${𝐳}_{\left(p,t\right)}^{\left(0\right)}=𝐄{𝐱}_{\left(p,t\right)}+{𝐞}_{\left(p,t\right)}^{\text{pos}}$$ (1)

**Fig 3:**
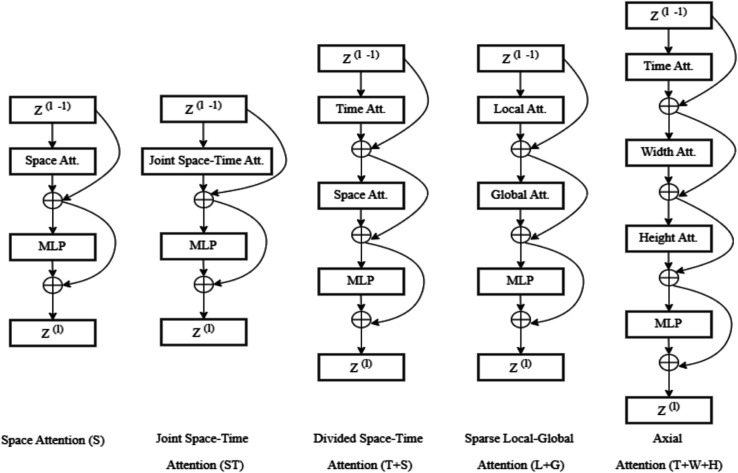
Five self-attention blocks of TimeSformer [56]. Each variant illustrates a different way of modeling spatial and temporal relationships in video data using transformer blocks. (1) Space Attention (S): attends only across spatial dimensions in each frame. (2) Joint Space-Time Attention (ST): computes attention jointly across space and time. (3) Divided Space-Time Attention (T+S): separates temporal and spatial attention sequentially. (4) Sparse Local-Global Attention (L+G): combines local and global spatial attention for broader context. (5) Axial Attention (T+W+H): factors attention across time, width, and height axes independently. Each block outputs updated video representations used for downstream tasks.

Self-attention improves computational efficiency by employing various attention techniques on patches, as demonstrated in Fig 3.

Spatial attention functions independently on a frame-by-frame basis, executing *N*  +  1 query-key comparisons. Joint space-time attention encompasses both spatial and temporal dimensions; nonetheless, it is computationally demanding, necessitating *NF*  +  1 comparisons, *F* denoting the total number of frames. Conversely, divided space-time attention successively analyzes temporal and spatial dimensions, attaining maximal accuracy with *N*  +  *F*  +  2 comparisons.

To improve efficiency, sparse local-global and axial attention equilibrate local and global emphasis while allocating attention across temporal, spatial width, and height dimensions. A multilayer perceptron with residual connections ultimately enhances the attention outputs.

### 3.5 Fine-tuning of video transformers

Fine-tuning is a process that adapts a pretrained classification model to a new task by retraining it on task-specific data [67]. The full configuration of training parameters is summarized in Table 2.

**Table 2:** Training hyperparameters.

Hyperparameter	Value
Training batch size	2
Evaluation batch size	2
Gradient accumulation steps	4
Total effective batch size	8
Initial learning rate	5e-5
Weight decay	0.01
Learning scheduler type	Linear
Warm-up ratio	0.1
Optimizer	AdamW
Loss Function	Cross entropy loss

The training process used the specified hyperparameters. We set the batch size to 2 for both training and evaluation, and accumulated gradients over 4 steps to obtain an effective batch size of 8. We used the AdamW optimizer with an initial learning rate of 5e-5, a linear scheduler, and a warm-up ratio of 0.1. To reduce overfitting, we applied a weight decay of 0.01, and used cross-entropy loss for classification.

In this study, we fine-tuned video transformer models on the BdSLW60 dataset to classify isolated Bangladeshi Sign Language (BdSL) words. Leveraging the Hugging Face Transformers library, we initialized models with pretrained weights, modified the classification head to match the number of target classes, and utilized previously learned features to enhance generalization.

To further improve performance, we integrated a task-specific classification head into the final layer and systematically tuned key hyperparameters, including batch size, learning rate, and weight decay. A dynamic learning rate scheduler was employed to adjust learning rates throughout training, while model weights were optimized via backpropagation. To mitigate overfitting, an early stopping mechanism was implemented, enabling the training process to terminate when performance plateaued on the validation set.

### 3.6 Model evaluation

Evaluating the performance of ML and DL models is crucial for both model development and deployment in real-world applications. Key evaluation metrics include accuracy, precision, recall, and the F1 score, each providing unique insights into model behaviour. Precision quantifies the proportion of correctly identified positive predictions, while accuracy measures the overall correctness across all classes. Recall evaluates the model’s ability to detect true positive instances, and the F1 score provides a harmonic mean of precision and recall, offering a balanced measure of classification performance [68].

To comprehensively assess the fine-tuned transformer models, we computed accuracy, precision, recall, and F1 scores on the test datasets. In addition, confusion matrices were generated to visualize class-wise performance and identify potential misclassifications. The loss curve was also analyzed throughout training to monitor convergence behavior and detect signs of overfitting.

## 4 Result analysis

The primary objective of this study was to identify the most effective pretrained video transformer model for classifying isolated BdSL signs using the BdSLW60 dataset, along with additional benchmark datasets.

Notably, this work also presents the first benchmarking results for the BdSLW401 dataset, contributing a valuable reference point for future research in BdSL recognition. To evaluate the impact of data augmentation on model performance, we examined two video preprocessing strategies: one incorporating augmentation techniques such as random horizontal flipping and cropping, and another that excluded such modifications.

The training hyperparameters were kept consistent across all experiments to ensure fair comparison. Table 3 summarizes the dataset partitioning strategies employed for training, validation, and testing. To support subject-independent evaluation, we implemented user-specific splits for BdSLW60, designating users U4 and U8 for testing and U5 for validation. For the LSA64 dataset, signer IDs 001 and 002 were assigned to the test set, while 10% of the remaining samples were used for validation.

**Table 3:** Dataset splitting configurations.

Dataset	Train	Test	Val
BdSLW60 [16]	7431	1276	600
BdSLW60 (10-fold) [16]	8031	1276	-
BdSLW401 Front [69]	38876	7833	4389
WLASL100 [70]	1442	258	338
WLASL2000 [70]	14289	2878	3916
LSA64 [11]	2304	640	256

The table lists the number of training, testing, and validation samples for each dataset. Some datasets, such as BdSLW60, include both the original splits and a 10-fold cross-validation setup. The counts vary substantially across datasets, reflecting differences in scale and experimental design.

In the case of BdSLW401, we utilized the front-view subset and adhered to its original train/validation/test split, where users S04 and S08 were reserved for testing. For WLASL100 and WLASL2000, we adopted the official JSON-based partitions. Additionally, to assess model robustness, we conducted experiments using a 10-fold stratified version of the BdSLW60 dataset.

### 4.1 Training approaches

Training was conducted using computational resources comprising a 32GB GPU and 164GB of CPU memory, enabling efficient processing across both large- and small-scale datasets. Models trained with data augmentation consistently outperformed those trained without it. Validation results using VideoMAE, ViViT and TimeSformer (Table 4) confirmed that augmentation led to improved test accuracy, surpassing prior benchmarks such as those in [16], which employed SVM and attention-based Bi-LSTM architectures. Despite BdSLW60 offering more samples per gloss, its inherent class imbalance negatively affected model accuracy. This issue was partially addressed through stratified K-fold cross-validation, which preserved the original class distribution across all folds, thereby enhancing evaluation robustness. Additionally, the use of 16-level relative quantization (RQ) in VideoMAE resulted in reduced accuracy, indicating a potential limitation of quantization-based compression in this context.

**Table 4:** Performance of VideoMAE, ViViT and TimeSformer with and without augmentation.

Model	Epoch	Test Accuracy	
		Aug: Yes	Aug: No
“MCG-NJU/videomae-base”	20	84.95%	69.59%
“MCG-NJU/videomae-base-finetuned-kinetics”	20	**92.95%**	91.54%
“google/vivit-b-16x2-kinetics400”	20	80.3%	74.37%
“MCG-NJU/videomae-base” with RQ	20	82.05%	70.61%
“facebook/timesformer-base-finetuned-k400”	20	77.6%	76.3%

It presents the test accuracy of VideoMAE, ViViT, and TimeSformer models evaluated with and without data augmentation after 20 training epochs. Data augmentation consistently improves performance across all models. The finetuned VideoMAE model (“MCG-NJU/videomae-base-finetuned-kinetics”) achieves the highest performance, reaching 92.95% accuracy and 98.90% top-5 accuracy with augmentation, and 91.54% accuracy without augmentation. Introducing relative quantization (RQ) to the VideoMAE base model results in a slight decrease in accuracy compared to the base model without RQ.

### 4.2 Comparing results among existing datasets

To enhance the validation of model performance, we extended our evaluation to include two public benchmark datasets: LSA64 [11] and WLASL [70]. Our primary focus, however, remained on isolated Bangla Sign Language recognition, using two datasets: BdSLW60 and BdSLW401. On the smaller-scale BdSLW60 dataset, Our transformer-based approach achieved an accuracy of 96.9% and a top-5 accuracy of 99.05%, surpassing existing baselines and demonstrating the model’s strong capability in recognizing isolated signs.

On the more extensive BdSLW401 dataset, which comprises 401 Bangla sign words, the model attained an accuracy of 81.04% after just 20 training epochs using the “MCG-NJU/videomae-base-finetuned-kinetics” architecture. This result is particularly promising given the dataset’s complexity and the prolonged training time of over five days. To further benchmark the generalization capacity of our approach, we evaluated performance on LSA64, WLASL100, and WLASL2000. Our model demonstrated improvements over previous deep learning methods on both LSA64 and WLASL100. However, performance on WLASL2000 was comparatively limited, which may be due to the highly imbalanced and sparse distribution of samples across its 2000 classes.

Table 5 summarizes the performance across the BdSLW and LSA64 datasets and presents Part 1 of our experimental evaluation, with boldface used to highlight the highest accuracies for each dataset.

**Table 5:** Experimental results on different models and datasets - Part 1.

Papers	Dataset	Model	Test Metrics			
			Acc	Pre	Rec	F1
Our Work	BdSLW60	“MCG-NJU/videomae-base”	93.6%	94.3%	93.7%	93.6%
		“MCG-NJU/videomae-base-finetuned-kinetics”	**96.9%**	97.2%	97.0%	96.9%
		“google/vivit-b-16x2-kinetics400”	81.0%	84.9%	81.0%	80.7%
		“facebook/timesformer-base-finetuned-k400”	82.1%	86.2%	82.1%	81.9%
	BdSLW401	“MCG-NJU/videomae-base-finetuned-kinetics”	**81.04%**	84.57%	81.14%	80.14%
	LSA64	“MCG-NJU/videomae-base”	96.25%	96.8%	96.3%	96.1%
		“MCG-NJU/videomae-base-finetuned-kinetics”	97.65%	98.3%	97.6%	97.5%
		“google/vivit-b-16x2-kinetics400”	98.9%	99.2%	98.9%	98.9%
		“facebook/timesformer-base-finetuned-k400”	**99.06%**	99.2%	99.1%	99.1%
BdSLW60 [16]	BdSLW60	SVM Attention-based bi-LSTM	67.6% **75.1%**			
LSA64 [11]	LSA64	“HMM-GMM”	95.95%			
3DGCN [71]	LSA64	“3D Graph Convolutional Neural Network”	94.84%			
HWGAT [72]	LSA64	“Hierarchical Windowed Graph Attention Network”	**98.59%**			

This table reports performance metrics across three dataset splits from two major isolated sign language recognition benchmarks: BdSLW (BdSLW60 and BdSLW401) and LSA64. We present accuracy (Acc), precision (Pre), recall (Rec), and F1-score (F1). Our finetuned VideoMAE model consistently demonstrates superior performance across these datasets. Prior results from the original studies are included for comparison. For each dataset, the highest accuracy among models is highlighted in bold.

Table 6 presents Part 2 of our experiments, detailing the performance of our finetuned models on the WLASL dataset. We conducted experiments on WLASL for both 20 and 200 training epochs. Additionally, we evaluated “MCG-NJU/videomae-base” for 40 and 50 epochs and “MCG-NJU/videomae-base-finetuned-kinetics” for 30 epochs on WLASL100, reporting all results.

Our findings indicate that the pretrained and finetuned models, except for the VideoMAE base variant, tend to converge rapidly in the early epochs but experience a performance drop at higher epoch counts due to overfitting. Furthermore, we surpass the results reported in [21] while using a batch size of 2, whereas their experiments utilized batch sizes of 4 and 6.

**Table 6:** Experimental results on different models and datasets - Part 2.

Papers	Dataset	Model	Epoch	Test Metrics					
				Top-1	Top-5	Top-10	Macro-P	Macro-R	Macro-F1
Our Work	WLASL 100	“MCG-NJU/videomae-base”	20	15.11%	39.92%	58.91%	14.78%	15.49%	13.76%
		“MCG-NJU/videomae-base”	40	32.55%	65.89%	78.29%	35.11%	32.11%	30.65%
		“MCG-NJU/videomae-base”	50	37.20%	67.44%	79.84%	39.10%	36.9%	34.79%
		“MCG-NJU/videomae-base-finetuned-kinetics”	20	**77.90%**	**93.79%**	**94.96%**	**80.55%**	**77.71%**	**76.62%**
		“MCG-NJU/videomae-base-finetuned-kinetics”	30	74.80%	92.24%	94.96%	76.58%	74.71%	73.05%
		“google/vivit-b-16x2-kinetics400”	20	65.50%	86.43%	90.69%	68.35%	65.85%	64.01%
		“facebook/timesformer-base-finetuned-k400”	20	67.44%	86.43%	92.24%	68.51%	68.29%	65.55%
		“MCG-NJU/videomae-base”	200	43.79%	72.86%	80.62%	49.32%	44.25%	42.13%
		“MCG-NJU/videomae-base-finetuned-kinetics”	200	67.44%	90.31%	96.12%	69.26%	67.59%	65.15%
		“google/vivit-b-16x2-kinetics400”	200	59.68%	84.49%	89.53%	66.48%	59.98%	59.05%
		“facebook/timesformer-base-finetuned-k400”	200	55.03%	79.84%	86.43%	55.54%	56.18%	51.96%
	WLASL 2000	“MCG-NJU/videomae-base”	20	0.27%	0.48%	0.83%	0.06%	0.26%	0.08%
		“MCG-NJU/videomae-base-finetuned-kinetics”	20	**7.08%**	**13.79%**	**15.63%**	**5.9%**	**6.72%**	**5.97%**
		“google/vivit-b-16x2-kinetics400”	20	5.8%	12.3%	14.4%	4.36%	5.45%	4.54%
		“MCG-NJU/videomae-base”	200	2.88%	7.4%	9.6%			
		“MCG-NJU/videomae-base-finetuned-kinetics”	200	6.9%	12.4%	14.5%			
		“google/vivit-b-16x2-kinetics400”	200	4.7%	10.4%	12.7%			
WLASL [70]	WLASL 100	Pose-GRU	200	46.51%	76.74%	85.66%			
		Pose-TGCN	200	55.43%	78.68%	87.60%			
		VGG-GRU	200	25.97%	55.04%	63.95%			
		I3D	200	**65.89%**	**84.11%**	**89.92%**			
	WLASL 2000	Pose-GRU	200	22.54%	49.81%	61.38%			
		Pose-TGCN	200	23.65%	51.75%	62.24%			
		VGG-GRU	200	8.44%	23.58%	32.58%			
		I3D	200	**32.48%**	**57.31%**	**66.31%**			
WLASL [21]	WLASL 100	VideoMAE	30	**75.58**%	**91.86%**	**95.74%**			
		TimeSformer	15	62.02%	87.98%	94.19%			

This table provides an overview of performance metrics across two dataset splits from WLASL (WLASL100 and WLASL2000). We present Top-1, Top-5, and Top-10 accuracies, along with Macro Precision (Macro-P), Macro Recall (Macro-R), and Macro F1-score (Macro-F1). Our finetuned VideoMAE model consistently achieves superior performance on WLASL100, while exhibiting performance degradation on the long-tailed WLASL2000 split. Prior results from the original studies are included for comparison. For each dataset, the highest test metric among the models is highlighted in bold.

**Fig 4:**
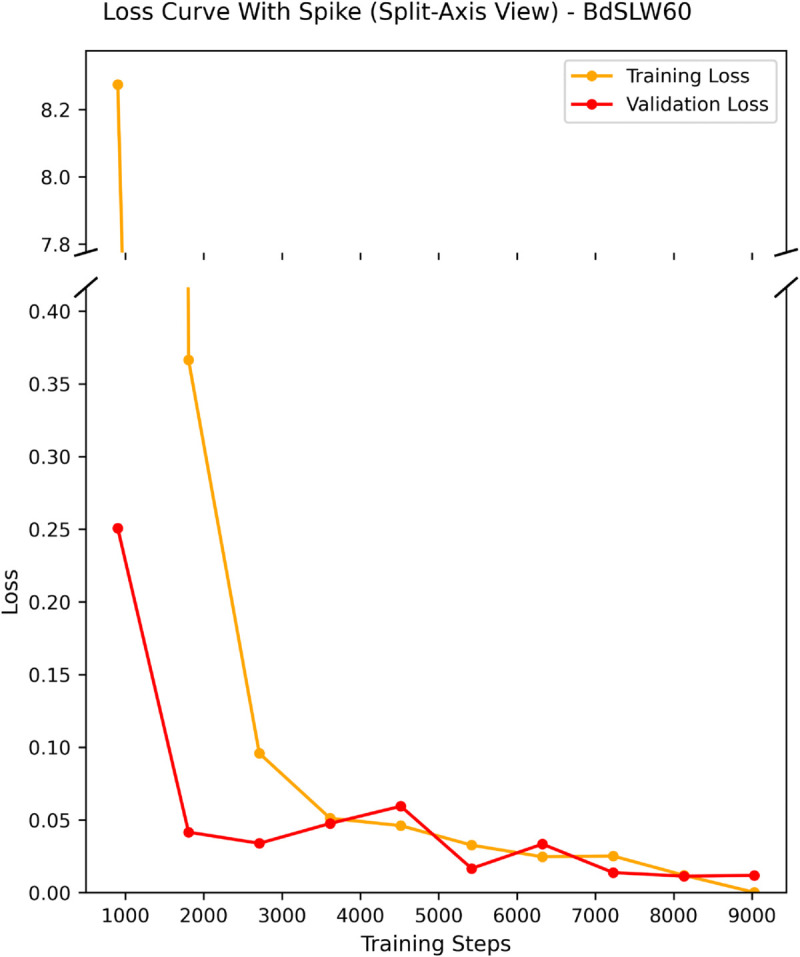
Loss curve for fold 6 of the BdSLW60 dataset. This figure shows the training and validation loss curves on BdSLW60 using a split-axis visualization to highlight the initial loss spike and subsequent convergence. After a brief early spike, both losses decrease rapidly and remain closely aligned, indicating stable learning, minimal overfitting, and good generalization throughout training.

The loss curve and confusion matrices offer additional insights into training behavior and classification performance. On BdSLW60, the loss curve (Fig 4) demonstrates rapid convergence followed by stability after approximately 2000 training steps, suggesting effective learning and strong generalization. The validation loss exhibits a slight spike early in the training process but quickly stabilizes, indicating minimal overfitting and a well-generalized model.

**Fig 5:**
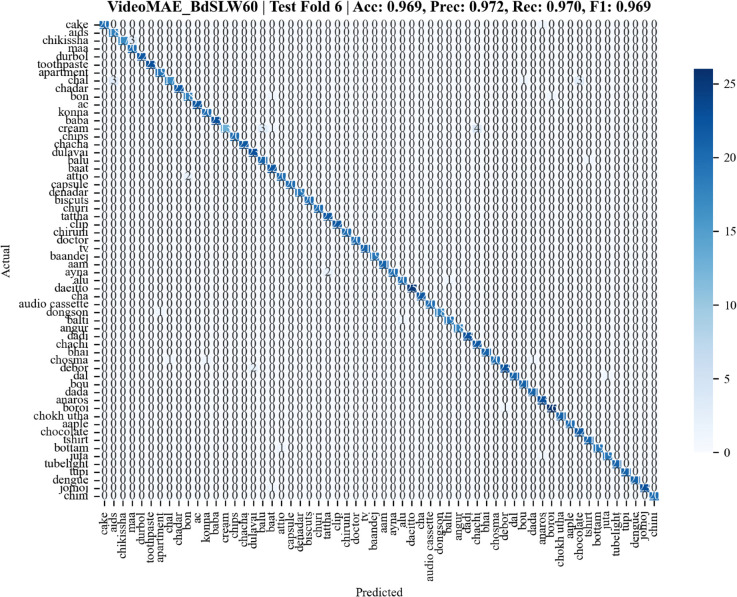
Confusion matrix for fold 6 of the BdSLW60 test set. This confusion matrix shows how well the model classified each of the 60 sign language gestures. The diagonal cells represent correct predictions, with darker shades indicating better performance. Most predictions fall along this diagonal, showing that the model accurately recognized the majority of signs. The few lighter cells outside the diagonal indicate occasional misclassifications, but overall, the results suggest strong and consistent performance across all classes.

**Fig 6:**
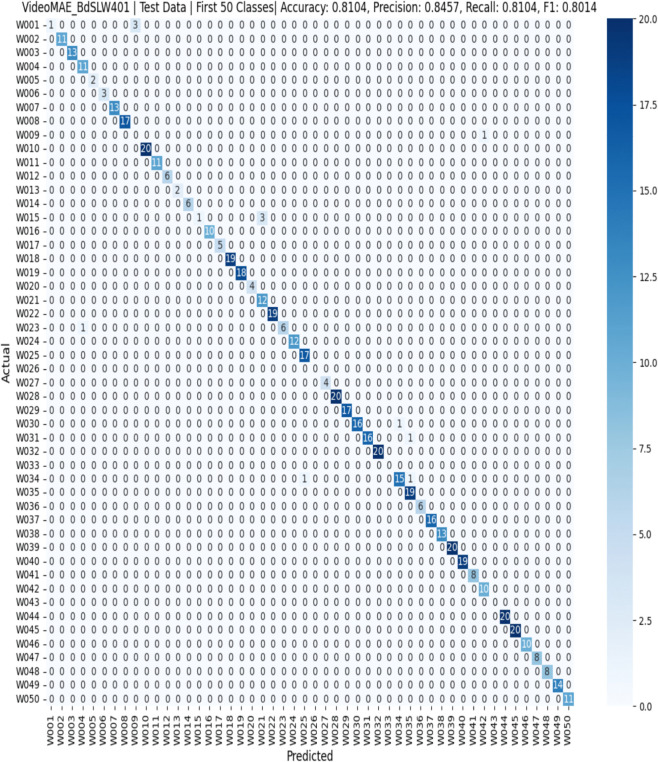
Confusion matrix for BdSLW401 test set (visualizing first 50 classes). This confusion matrix presents the model’s classification results for the first 50 classes out of a total of 401 in the BdSLW401 test set. Each row represents the actual class label, while each column shows the predicted label. The darker diagonal cells indicate correct predictions, suggesting the model has learned to recognize many of the signs accurately within this subset. The few lighter off-diagonal entries represent misclassifications, pointing to some confusion between certain signs. This visualization provides insight into the model’s performance on a portion of the full class set.

The confusion matrix for BdSLW60 (Fig 5) confirms accurate class-wise predictions, while Fig 6 illustrates the confusion matrix for BdSLW401, demonstrating the model’s ability to scale effectively to a larger vocabulary.

Together, these comparisons underscore the robustness and adaptability of transformer-based video models across both low- and high-resource sign language datasets.

### 4.3 Ablation studies

To optimize computational efficiency on our system, a batch size of two was employed. Larger batch sizes were avoided due to the associated increase in memory and storage requirements. During training, the entire pretrained model was initialized, and all layers were fine-tuned on our task-specific datasets. A learning rate scheduler dynamically adjusted the learning rate post-initialization, while the AdamW optimizer with decoupled weight decay—was used to mitigate overfitting, making it particularly effective for large-scale model fine-tuning despite substantial memory demands.

We applied Frame Rate Correction (FRC) to BdSLW60 clips originally recorded at 15 and 24 FPS, converting them to 30 FPS for consistency. However, this led to frame duplication, which negatively affected generalization by distorting attention patterns and gradient updates. As a result, VideoMAE showed reduced accuracy on FRC clips compared to uncorrected samples. To address this, we introduced variability into the duplicated frames through random flipping, cropping, and scaling during preprocessing. This enhancement consistently improved performance, as shown in Table 4.

Frame sampling rate played a crucial role in ensuring reliable and consistent input across clips, as detailed in Table 7. For instance, WLASL2000 was trained for 200 epochs, with most outputs stabilizing after 40–50% of the training cycle.

**Table 7:** Dataset details with FPS, models, SR and clip durations.

Dataset	FPS	Model	Sample rate (SR)	Clip Duration
BdSLW60	30	ViViT	4	4.27 s
		VideoMAE	8	
		Timesformer	16	
BdSLW401	30	ViViT	5	5.34 s
		VideoMAE	10	
		Timesformer	20	
LSA64	60	ViViT	6	3.2 s
		VideoMAE	12	
		Timesformer	24	
WLASL	25	ViViT	4	5.2 s
		VideoMAE	8	
		Timesformer	16	

This table summarizes the frame rates (FPS), model-specific sampling rates (SR), and resulting clip durations for each dataset and model. The three models ViViT, VideoMAE, and TimeSformer require different frame sampling configurations based on their architectural designs. To maintain consistency within each dataset, we adjusted the sampling rates such that all models process clips of the same temporal length. However, due to variations in the native FPS and video characteristics of the datasets, the resulting clip durations differ across datasets.

We construct a correlation table to analyze the relationship between classification accuracy and several class-wise factors, including average temporal length (number of frames), number of samples, and signer identity. The analysis reveals that classification accuracy varies across signers for all evaluated models and datasets, while the remaining factors show no, weak, or moderate levels of association with performance. The resulting correlation statistics are summarized in Table 8.

**Table 8:** Correlation summary across datasets and models.

Dataset	Model	Accuracy vs Class Frequency	Accuracy vs Avg. Frames per Class	Accuracy vs Unique Signers per Class
BdSLW60	“MCG-NJU/videomae-base-finetuned-kinetics”	No	No	No
	“google/vivit-b-16x2-kinetics400”	No	No	No
	“facebook/timesformer-base-finetuned-k400”	No	No	No
LSA64	“google/vivit-b-16x2-kinetics400”	No	No	No
WLASL 100	“MCG-NJU/videomae-base”	No	Yes - moderate (+ve)	No
	“MCG-NJU/videomae-base-finetuned-kinetics”	Yes - weak (-ve)	Yes - moderate (+ve)	No
	“google/vivit-b-16x2-kinetics400”	No	No	No
	“facebook/timesformer-base-finetuned-k400”	No	No	Yes - weak (-ve)
WLASL 2000	“MCG-NJU/videomae-base”	Yes - moderate (+ve)	Yes - very weak (+ve)	Yes - weak (+ve)
	“MCG-NJU/videomae-base-finetuned-kinetics”	Yes - moderate (+ve)	Yes - weak (+ve)	Yes - weak (+ve)
	“google/vivit-b-16x2-kinetics400”	Yes - moderate (+ve)	Yes - weak (+ve)	Yes - weak (+ve)

This table summarizes the relationships between model accuracy and three dataset-level factors—class frequency, average frames per class, and unique signers per class—across multiple datasets (BdSLW60, LSA64, WLASL100, and WLASL2000) and pretrained video models (VideoMAE, ViViT, and TimeSformer). Entries indicate the presence, strength, and direction of correlations. Overall, little to no correlation is observed for BdSLW60 and LSA64, whereas highly imbalanced datasets (WLASL100 and WLASL2000) show weak to moderate correlations, with VideoMAE showing greater sensitivity to data scale and signer diversity than ViViT and TimeSformer.

Among the evaluated models, TimeSformer achieved a high accuracy of 99.06% on the LSA64 dataset. Its superior performance is attributed to LSA64’s balanced class distribution, 60 FPS recording rate, and clip lengths ranging between 90 and 180 frames.

We standardized each clip to 3.2 seconds (192 frames), applying a sampling rate of 24 to extract 8 frames per video. Extending shorter clips to match this duration helped retain critical temporal information. In contrast, BdSLW60, frame rate corrected to 30 FPS with imbalanced class distribution, posed additional challenges. Given that 99% of BdSLW60 clips contain fewer than 128 frames, we extracted 4.27-second clips for uniformity.

Although BdSLW60 exhibits variable clip lengths (9–164 frames), the average frame count does not correlate with classification accuracy. This is likely due to the sufficient number of samples per class, with averages of 21 test samples and 10 validation samples, a trend further supported by the weak correlation observed when evaluating the TimeSformer model on BdSLW60 without data augmentation or a folding approach.

In contrast, WLASL100 contains only about three samples per class, where uniform temporal subsampling with limited data leads to a moderate correlation between average frame count and per-class accuracy. However, this correlation is very weak in WLASL2000, as larger class sizes and more uniform temporal lengths across classes reduce the impact of frame count on performance.

Due to the sufficient number of samples across all classes and the limited variation of unique signers (12-15) among classes in the training set, no relationship is observed between class frequency or the number of unique signers and classification accuracy. A similar trend is observed in the LSA64 dataset.

On BdSLW60, VideoMAE achieved superior performance by sampling 16 frames at an 8-frame rate and employing a high-ratio masking strategy that retains only 10% of tokens for encoding while reconstructing the remaining 90% using mean squared error. This approach enables effective preservation of essential spatiotemporal features, whereas ViViT and TimeSformer exhibit comparatively lower accuracy.

No long-tail effect is observed in the WLASL100 and BdSLW60 datasets. In WLASL100, the VideoMAE-Kinetics model shows a weak negative correlation between class accuracy and class frequency; however, this correlation is negligible and does not indicate a meaningful long-tail behavior.

**Fig 7:**
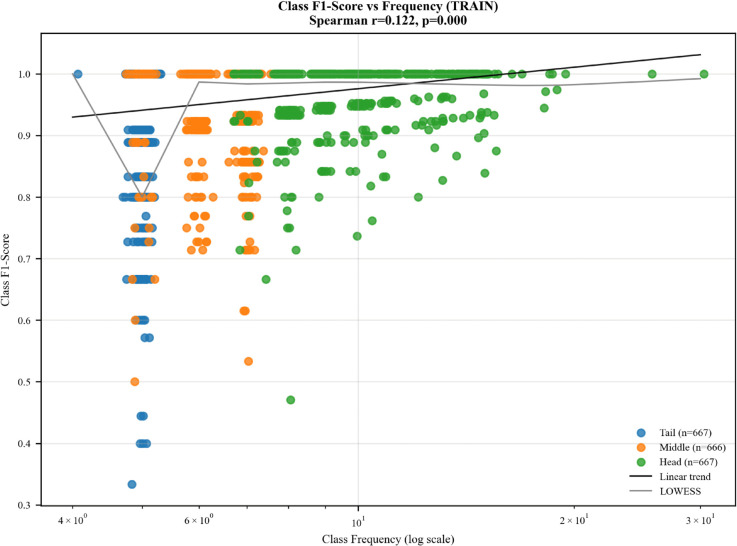
Long-tail nature of WLASL-2000 on VideoMAE kinetics model. This figure illustrates the long-tail effect in WLASL-2000, showing class-wise F1-score versus training-set class frequency (log scale), grouped into head, middle, and tail classes. A weak but statistically significant positive correlation is observed (Spearman *r* = 0.122, *p* < 0.001), with higher variability among tail classes.

In contrast, the WLASL2000 dataset exhibits a clear long-tail effect, as shown in Fig 7. This behavior is consistently observed across all evaluated models, although we present results only for the VideoMAE kinetics model for clarity. The model performs strongly on frequent classes but struggles with rare ones, and increasing class frequency particularly for tail classes generally leads to improved F1-scores.

Additionally, WLASL2000 contains between 2 and 18 unique signers per class in the training set, and we observe a slight accuracy gain for classes with a higher number of unique signers, suggesting that signer diversity contributes modestly to improved generalization. The WLASL2000 dataset presented additional challenges, including severe class imbalance, and small per-class sample sizes, all of which contributed to degraded performance. These findings suggest that transformer-based video models perform optimally when trained on datasets without long-tail effects and with sufficient class representation.

## 5 Conclusion and future work

Sign language recognition plays a vital role in reducing communication barriers between deaf and hearing communities by translating visual gestures, including hand and facial expressions, into meaningful language.

To evaluate the robustness and scalability of our approach, we carried out extensive experiments using three different video transformer architectures across multiple sign language datasets, with fine-tuning initiated on BdSLW60 as our primary data source before extending to larger and more diverse benchmarks. The raw videos were first standardized to a uniform frame rate and segmented into shorter clips for effective training, followed by data augmentation strategies including random cropping, flipping, and scaling to enhance model generalization.

Using BdSLW60, the MCG-NJU/videomae-base-finetuned-kinetics model achieved a test accuracy of 96.9%, demonstrating strong performance in isolated BdSL recognition. To further evaluate model scalability, we introduce the first benchmark results on the BdSLW401 dataset, a larger and more diverse collection of 401 Bangla signs. On this dataset, the model achieved 81.04% accuracy, with an F1 score of 80.14%, recall of 84.57%, and precision of 81.14%.

Additional experiments on LSA64 and WLASL showed that factors such as frame distribution, sample size, and architecture significantly affect recognition accuracy. Overall, our approach outperforms prior methods in Bangla word-level sign language recognition. Future work will extend to sentence-level BdSL recognition and real-time translation applications.

## Supporting information

S1 File List of trained checkpoints links. This pdf file contains the URLs of all best-validation checkpoints hosted on Hugging Face that were obtained in this research.
(PDF)

S1 Fig Correlation between accuracy and average frames on WLASL-2000 (ViViT Model). (TIFF)

S2 Fig Correlation between accuracy and class sample size on WLASL-2000 (ViViT Model). (TIFF)

S3 Fig Correlation between accuracy and number of unique signers on WLASL-2000 (ViViT Model). (TIFF)

S4 Fig Accuracy per signer ID on WLASL-2000 (ViViT Model). (TIFF)

S5 Fig Correlation between accuracy and average frames on WLASL-100 (VideoMAE Model). (TIFF)

S6 Fig Correlation between accuracy and class sample size on WLASL-100 (VideoMAE Model). (TIFF)

S7 Fig Correlation between accuracy and number of unique signers on WLASL-100 (TimeSformer Model). (TIFF)

S8 Fig Correlation between accuracy and average frames on BdSLW60 (TimeSformer Model) with augmentation. (TIFF)

S9 Fig Correlation between accuracy and average frames on BdSLW60 (TimeSformer Model) without augmentation. (TIFF)

S10 Fig Correlation between accuracy and average frames on LSA64 (ViViT Model). (TIFF)
