# Supplementary material for: A comparative analysis of video vision transformers on word-level sign language datasets
Source: PLoS One. 2026 Feb 5;21(2):e0341909. doi: 10.1371/journal.pone.0341909 (PMC12875579; doi:10.1371/journal.pone.0341909)
Supplement: S1 File — This pdf file contains the URLs of all best-validation checkpoints hosted on Hugging Face that were obtained in this research. (PDF) [file pone.0341909.s001.pdf]

Trained Checkpoints

The best-performing validation checkpoints from our experiments are publicly available on Hugging Face to support reproducibility and further research, and can be accessed via the following links.

BDSLW60

1. https://huggingface.co/Shawon16/VideoMAE_BdSLW60_FrameRate_Corrected_with_Augment_20_epoch_val_U5
2. https://huggingface.co/Shawon16/VideoMAE_BdSLW60_FrameRate_Corrected_withOUT_Augment_20_epoch_val_U5_again
3. https://huggingface.co/Shawon16/videoMAE_BDSLW60_U5_20_coR_new
4. https://huggingface.co/Shawon16/VideoMAE_BdSLW60_SR_8_kineticsFinetuned_withoutAug
5. https://huggingface.co/Shawon16/ViViT_BdSLW60_FrameRate_Corrected_with_Augment_20_epch
6. https://huggingface.co/Shawon16/ViViT_BdSLW60_FrameRate_Corrected_without_Augment_20_epch
7. https://huggingface.co/Shawon16/VideoMAE_BdSLW60_FrameRate_Corrected_WIT_Augment_20_epoch_RQ_
8. https://huggingface.co/Shawon16/VideoMAE_BdSLW60_FrameRate_Corrected_WITHOUT_Augment_20_epoch_RQ_GB
9. https://huggingface.co/Shawon16/timesformer_BDSLW60_U5_20_coR
10. https://huggingface.co/Shawon16/Timesformer_BDSLW60_U5_20_coR_withoutAug
11. https://huggingface.co/Shawon16/VideoMAE_Kinetics_fold__5__BdSLW60_SKF
12. https://huggingface.co/Shawon16/VideoMAE_default_fold__0__10_epoch_Aug_batch_1_4_BdSLW60
13. https://huggingface.co/Shawon16/Timesformer_default_fold__2__10_epoch_Aug_batch_2_4_BdSLW60
14. https://huggingface.co/Shawon16/ViViT_default_fold__3__10_epoch_Aug_batch_2_4_BdSLW60

BDSLW401

1. https://huggingface.co/Shawon16/VideoMAE_BdSLW401_20_epochs_p5_SR_10

LSA64

1. https://huggingface.co/Shawon16/VideoMAE_LSA64_SR_12
2. https://huggingface.co/Shawon16/VideoMAE_LSA64SR_8_kineticsFinetuned
3. https://huggingface.co/Shawon16/ViViT_lsa64_coR
4. https://huggingface.co/Shawon16/Timesformer_LSA64_SR_24

WLASL 100

1. https://huggingface.co/Shawon16/videoMAE_kinetics_wlasl_100__signer_20ep_coR
2. https://huggingface.co/Shawon16/videoMAE_kinetics_wlasl100_coR_30ep
3. https://huggingface.co/Shawon16/ViViT_wlasl_100__signer_20ep_coR_
4. https://huggingface.co/Shawon16/timesformer_wlasl_100_20ep_coR_
5. https://huggingface.co/Shawon16/VideoMAE_base_wlasl_100__signer_20ep_coR
6. https://huggingface.co/Shawon16/videoMAE_base_wlasl_100_40ep_coR_p10
7. https://huggingface.co/Shawon16/videoMAE_base_wlasl_100_50ep_coR_p10
8. https://huggingface.co/Shawon16/videoMAE_kinetics_wlasl_100__signer_200ep_coR
9. https://huggingface.co/Shawon16/ViViT_wlasl_100_200ep_coR_
10. https://huggingface.co/Shawon16/timesformer_wlasl_100_200ep_coR_
11. https://huggingface.co/Shawon16/VideoMAE_base_wlasl_100__signer_200ep_coR

WLASL 2000

1. https://huggingface.co/Shawon16/VideoMAE_WLASL_2000_200_epochs_p20_SR_8_kinetics
2. https://huggingface.co/Shawon16/ViViT_WLASL_200_epochs_p20
3. https://huggingface.co/Shawon16/VideoMAE_WLASL_250_epochs
4. https://huggingface.co/Shawon16/videomae_base_wlasl_2000_20ep_coR
5. https://huggingface.co/Shawon16/videomae_kinetics_wlasl_2000_20ep_coR
6. https://huggingface.co/Shawon16/ViViT_wlasl_2000_20ep_coR
